# Supplementary material for: Wastewater-based epidemiology for comprehensive communitywide exposome surveillance: A gradient of metals exposure
Source: medRxiv. 2023 Sep 29:2023.09.26.23295844. Preprint. [Version 1] doi: 10.1101/2023.09.26.23295844 (PMC10557802; doi:10.1101/2023.09.26.23295844)
Supplement: Supplement 1 [file NIHPP2023.09.26.23295844v1-supplement-1.pdf]

medRxiv preprint doi: https://doi.org/10.1101/2023.09.26.23295844; this version posted September 29, 2023. The copyright holder for this preprint (which was not certified by peer review) is the author/funder, who has granted medRxiv a license to display the preprint in perpetuity.
It is made available under a CC-BY 4.0 International license .

Supplementary Material

Wastewater-based epidemiology for comprehensive communitywide exposome surveillance: A gradient of metals exposure

Lu Cai[1], Rochelle H. Holm[2]*, Donald J. Biddle[3], Charlie H. Zhang[4], Daymond Talley[5], Ted Smith[2#], J. Christopher States[6#]*

[#]Joint senior authors

*Joint corresponding authors

Rochelle H. Holm, Christina Lee Brown Envirome Institute, School of Medicine, University of Louisville, 302 E. Muhammad Ali Blvd., Louisville, KY 40202, United States; rochelle.holm@louisville.edu

J. Christopher States, Department of Pharmacology and Toxicology, Center for Integrative Environmental Health Sciences, University of Louisville, 505 S. Hancock St. Rm 304, Louisville, KY 40202; jcstates@louisville.edu

Addresses:

[1]Department of Pediatrics, Pediatrics Research Institute, Center for Integrative Environmental Health Sciences, University of Louisville, Louisville, KY

[2]Christina Lee Brown Envirome Institute, School of Medicine, University of Louisville, 302 E. Muhammad Ali Blvd., Louisville, KY 40202, United States

[3]Department of Geographic and Environmental Sciences, Center for Geographic Information Sciences, University of Louisville, Louisville, KY

[4]Department of Geographic and Environmental Sciences, Center for Integrative Environmental Health Sciences, University of Louisville, Louisville, KY

[5]Morris Forman Water Quality Treatment Center, Louisville, KY, USA

[6]Department of Pharmacology and Toxicology, Center for Integrative Environmental Health Sciences, University of Louisville, Louisville, KY

medRxiv preprint doi: https://doi.org/10.1101/2023.09.26.23295844; this version posted September 29, 2023. The copyright holder for this preprint (which was not certified by peer review) is the author/funder, who has granted medRxiv a license to display the preprint in perpetuity.

It is made available under a CC-BY 4.0 International license .

Table of Contents

Table S1. Metals studied by Inductively Coupled Plasma Quadrupole Mass Spectrometer, detection limit, and standard curve levels. 43

Table S2. Metal concentrations of equipment rinse blank field sample. 44

Table S3. Mean metal concentrations by site. 45

medRxiv preprint doi: https://doi.org/10.1101/2023.09.26.23295844; this version posted September 29, 2023. The copyright holder for this preprint (which was not certified by peer review) is the author/funder, who has granted medRxiv a license to display the preprint in perpetuity.

It is made available under a CC-BY 4.0 International license .

Table S1. Metals studied by Inductively Coupled Plasma Quadrupole Mass Spectrometer, detection limit, and standard curve levels.

Metal	Detection limit (ng/mL)	Standard curve level
Ag	0.002	0, 1, 2, 5, 10, 50 ng
AI	0.357	0, 1, 2, 5, 10, 50 ng
As	0.007	0, 1, 2, 5, 10, 50 ng
Ba	0.008	0, 1, 2, 5, 10, 50 ng
Be	0	0, 1, 2, 5, 10, 50 ng
Ca	21.82	0, 100, 200, 500, 1000, 5000
Cd	0	0, 1, 2, 5, 10, 50 ng
Co	0.003	0, 1, 2, 5, 10, 50 ng
Cr	0.038	0, 1, 2, 5, 10, 50 ng
Cu	0.009	0, 1, 2, 5, 10, 50 ng
Fe	0.1	0, 100, 200, 500, 1000, 5000
K	8.31	0, 100, 200, 500, 1000, 5000
Mg	0.064	0, 100, 200, 500, 1000, 5000
Mn	0.043	0, 1, 2, 5, 10, 50 ng
Mo	0.005	0, 1, 2, 5, 10, 50 ng
Na	4.23	0, 100, 200, 500, 1000, 5000
Ni	0.031	0, 1, 2, 5, 10, 50 ng
Pb	0	0, 1, 2, 5, 10, 50 ng
Pt	0	0, 1, 2, 5, 10, 50 ng
Sb	0.003	0, 1, 2, 5, 10, 50 ng
Se	0.321	0, 1, 2, 5, 10, 50 ng
Th	0.001	0, 1, 2, 5, 10, 50 ng
TI	0.001	0, 1, 2, 5, 10, 50 ng
U	0.001	0, 1, 2, 5, 10, 50 ng
V	0.006	0, 1, 2, 5, 10, 50 ng
Zn	0.136	0, 1, 2, 5, 10, 50 ng

medRxiv preprint doi: https://doi.org/10.1101/2023.09.26.23295844; this version posted September 29, 2023. The copyright holder for this preprint (which was not certified by peer review) is the author/funder, who has granted medRxiv a license to display the preprint in perpetuity.

It is made available under a CC-BY 4.0 International license .

Table S2. Metal concentrations of equipment rinse blank field sample.

Metal	Concentration (ng/mL)
Ag	0.120
Al	235
As	0.467
Ba	40.347
Be	0.093
Ca	46103
Cd	0.053
Co	0.427
Cr	Below quantification
Cu	6.573
Fe	108.92
K	16837
Mg	16485
Mn	19.65
Mo	52.600
Na	68647
Ni	3.280
Pb	0.253
Pt	0.107
Sb	0.933
Se	2.347
Th	0.000
Tl	Below quantification
U	0.440
V	0.320
Zn	60.253

Table S3. Mean metal concentrations by site.

Sample #	Ag ng/mL	Al ng/mL	As ng/mL	Ba ng/mL	Be ng/mL	Ca ng/mL	Cd ng/mL	Co ng/mL	Cr ng/mL	Cu ng/mL	Fe ng/mL	K ng/mL	Mg ng/mL
E1	0.040	8580	1.093	53.147	0.000	27645	0.107	0.440	0.280	72.080	392.52	3967	11896
E2	0.040	142	0.773	31.293	0.093	54968	0.027	0.400	0.560	8.800	498.03	10835	13820
E3	0.040	53	1.760	40.453	0.000	63087	0.013	0.333	0.280	7.173	665.27	17957	22103
E4	0.280	151	1.027	60.613	0.187	48311	0.027	0.573	0.380	13.053	505.32	12648	16581
E5	0.227	80	1.027	37.467	0.280	53295	0.173	7.493	1.680	15.667	244.43	8199	18402
E7	0.027	110	0.973	27.613	0.093	37148	0.080	0.400	0.360	13.093	287.15	11495	12220
E8	0.067	212	1.040	399.413	0.000	41114	0.080	0.573	1.820	12.907	1154.79	14983	13506
E9	0.040	20355	1.200	54.147	1.680	63572	0.067	0.480	0.973	8.293	597.85	230955	16127
E10	0.067	56	0.653	48.840	0.280	72859	0.040	0.187	0.320	10.947	171.97	5203	14174
E11	0.107	120	0.453	42.987	0.000	66355	0.107	0.240		11.720	262.61	5075	12354
E12	0.040	19104	1.493	60.453	2.160	67386	0.093	0.467	1.400	34.187	866.28	261422	21387
E13	0.053	93	0.440	41.480	0.093	71186	0.080	0.280	5.787	10.400	598.20	6047	18655
E14	0.160	138	0.333	39.507	0.000	70766	0.120	0.427	1.800	29.587	297.44	9497	13500
E15	0.080	573	0.693	43.547	0.093	46049	0.040	0.653	1.520	16.413	509.61	20782	20317
E16	0.020	286	0.320	42.213	0.093	30519	0.027	0.533		3.453	99.39	20650	13063
E17	0.253	144	0.667	40.707	0.093	78044	0.067	0.227	0.040	13.853	173.47	9646	23073
E18	0.120	192	0.627	44.480	0.000	67560	0.480	0.573	0.240	32.667	509.69	9305	19137
E19	0.067	121	0.693	36.613	0.093	66923	0.027	0.267	0.520	12.453	226.56	11526	26593
E20	0.093	127	0.373	39.680	0.187	54919	0.053	0.267	0.040	16.853	195.85	10668	24562
E21	0.573	2766	1.147	74.667	0.187	59506	0.120	1.573	5.200	55.787	1295.69	10080	21922
E22	0.080	111	0.707	45.427	0.093	58811	0.027	0.320	0.360	14.747	271.15	11241	26226
E23	0.080	201	0.840	29.680	0.093	50687	0.067	0.373	1.600	23.627	657.33	16272	18862
E24	0.133	127	0.733	33.333	0.373	42384	0.053	0.347	5.200	23.947	421.32	13801	15209
E25	0.027	42	0.493	39.147	0.000	70539	0.040	0.333		11.880	480.37	16802	18333
E26	0.040	17394	1.560	29.347	1.880	45913	0.053	0.573	6.880	9.227	496.04	363041	16422
E27	0.147	1180	1.080	38.040	0.093	85790	0.120	1.133	1.373	26.267	754.63	36019	19356
E28	0.040	152	0.720	199.133	0.280	3410126	0.173	2.067		19.947	151.56	22609	71163

medRxiv preprint doi: https://doi.org/10.1101/2023.09.26.23295844; this version posted September 29, 2023. The copyright holder for this preprint (which was not certified by peer review) is the author/funder, who has granted medRxiv a license to display the preprint in perpetuity.
It is made available under a CC-BY 4.0 International license .

Sample #	Mn ng/mL	Mo ng/mL	Na ng/mL	Ni ng/mL	Pb ng/mL	Pt ng/mL	Sb ng/mL	Se ng/mL	Th ng/mL	Tl ng/mL	U ng/mL	V ng/mL	Zn ng/mL
E1	23.99	356.173	281538	87.387	0.707	0.120	0.187	5.000	0.133	0.000	0.133	0.507	15.453
E2	116.47	1.667	29330	4.027	1.613	0.133	0.253	2.640	0.107	0.000	0.187	0.427	38.053
E3	201.25	1.387	49938	6.547	0.787	0.133	0.187	3.960	0.040		0.240	0.213	26.347
E4	81.57	1.160	81944	6.107	1.427	0.133	0.320	4.860	0.027		0.307	0.440	36.907
E5	79.04	3.827	48540	219.093	1.027	0.120	0.533	1.760	0.200	0.360	0.347	0.547	35.173
E7	40.85	0.813	42983	1.640	1.867	0.133	0.333	6.180	0.000	0.000	0.453	0.427	28.213
E8	233.61	1.493	46298	2.787	4.227	0.120	0.640	4.720	0.000		0.493	7.680	46.013
E9	158.79	2.267	538474	1.693	0.813	0.387	0.333	4.420	0.027	0.020	0.467	1.573	78.413
E10	82.40	0.320	39067	1.240	0.773	0.107	0.133	6.620	0.000		0.453	0.280	33.547
E11	73.99	0.360	39920	1.467	2.680	0.120	0.147	2.200	0.000		0.413	0.307	43.227
E12	178.15	2.173	519013	2.787	1.253	0.293	0.507	1.320	0.013	0.000	0.493	1.880	54.600
E13	53.69	0.613	56266	2.493	1.173	0.107	0.187	4.400	0.067		0.440	0.493	397.387
E14	49.73	0.747	45989	2.320	2.027	0.107	0.200	2.200	0.040	0.060	0.467	0.440	79.733
E15	27.91	45.787	83557	4.707	3.240	0.133	0.787	2.053	0.013	0.000	0.413	0.493	115.667
E16	18.39	79.253	78686	4.320	0.133	0.133	1.173	1.320			0.347	0.267	57.933
E17	22.52	1.467	49306	1.147	0.373	0.093	0.320	3.080	0.000		0.467	0.347	72.520
E18	86.19	2.187	55692	3.160	2.587	0.093	0.240	2.200	0.000	0.000	0.493	0.613	70.893
E19	22.92	1.147	56050	1.400	0.400	0.093	0.333	2.200	0.000	0.000	0.547	0.307	52.040
E20	30.60	0.653	46599	1.907	0.880	0.120	0.373	3.960	0.000	0.000	0.600	0.320	64.333
E21	214.87	4.813	47839	12.213	5.480	0.107	0.827	0.880	0.040	0.027	1.133	1.013	175.400
E22	68.13	1.000	43767	1.720	1.200	0.093	0.307	3.520	0.000	0.000	0.493	0.427	60.560
E23	72.92	1.280	48357	2.733	8.800	0.080	0.520	5.280	0.027		0.267	0.453	75.800
E24	84.65	0.813	56362	3.347	2.093	0.093	0.267	3.980	0.000		0.293	0.267	56.613
E25	80.11	1.000	62629	4.800	0.587	0.093	0.213	3.520	0.000	0.000	0.427	0.360	27.120
E26	128.07	2.440	630457	2.973	1.653	0.147	0.320	3.080	0.000	0.000	0.467	2.960	40.253
E27	114.28	7.147	100994	12.120	3.320	0.160	0.480	1.760	0.053	0.000	0.427	0.987	75.787
E28	1038.72	8.040	1124647	7.613	1.053	0.840	0.960	3.227	0.000	0.013	0.707	0.960	66.133

medRxiv preprint doi: https://doi.org/10.1101/2023.09.26.23295844; this version posted September 29, 2023. The copyright holder for this preprint (which was not certified by peer review) is the author/funder, who has granted medRxiv a license to display the preprint in perpetuity.
It is made available under a CC-BY 4.0 International license .
